# Environmental exposures and health behavior in association with mental health: a study design

**DOI:** 10.1186/s13690-020-00477-0

**Published:** 2020-10-21

**Authors:** Pauline Hautekiet, Tim S. Nawrot, Stefaan Demarest, Johan Van der Heyden, Ilse Van Overmeire, Eva M. De Clercq, Nelly D. Saenen

**Affiliations:** 1 Sciensano, Brussels, Belgium; 2 Centre for Environmental Sciences, Hasselt University, Agoralaan gebouw D, BE-3590 Hasselt, Belgium; 3 Centre for Environment and Health, Leuven University, Leuven, Belgium

**Keywords:** Biomarkers, Air pollution, Green space, Smoking, Mental health, Biological aging, Belgium

## Abstract

**Background:**

Air pollution, green space and smoking are known to affect human health. However, less is known about their underlying biological mechanisms. One of these mechanisms could be biological aging. In this study, we explore the mediation of biomarkers of exposure and biological aging to explain the associations between environmental exposures, health behavior and mental health.

**Methods:**

The study population of this cross-sectional study (*n* = 1168) is a subsample of the Belgian 2018 Health Interview Survey (BHIS). Mental health indicators including psychological and severe psychological distress, life satisfaction, vitality, eating disorders, suicidal ideation, subjective health and depressive and anxiety disorders, demographics and health behavior such as smoking are derived from the BHIS. Urine and blood samples are collected to measure respectively the biomarkers of exposure (urinary black carbon (BC) and (hydroxy)cotinine) and the biomarkers of biological aging (mitochondrial DNA content (mtDNAc) and telomere length (TL)). Recent and chronic exposure (μg/m^3^) to nitrogen dioxide (NO_2_), particulate matter ≤2.5 μm (PM_2.5_) and ≤ 10 μm (PM_10_) and BC at the participants’ residence are modelled using a high resolution spatial temporal interpolation model. Residential green space is defined in buffers of different size (50 m – 5000 m) using land cover data in ArcGIS 10 software. For the statistical analysis multivariate linear and logistic regressions as well as mediation analyses are used taking into account a priori selected covariates and confounders.

**Results:**

As this study combined data of BHIS and laboratory analyses, not all data is available for all participants. Therefore, data analyses will be conducted on different subsets. Data on air pollution and green space exposure is available for all BHIS participants. Questions on smoking and mental health were answered by respectively 7829 and 7213 BHIS participants. For biomarker assessment, (hydroxy) cotinine, urinary BC and the biomarkers of biological aging are measured for respectively 1130, 1120 and 985 participants.

**Conclusion:**

By use of personal markers of air pollution and smoking, as well as biological aging, we will gain knowledge about the association between environmental exposures, health behavior, and the mental health status. The results of the study can provide insights on the health of the Belgian population, making it a nationwide interesting study.

## Background

Human health is determined by health behavior such as smoking and by exposures like residential green space and air pollution, including fine (≤ 2.5 μm (PM_2.5_)) and coarse (≤ 10 μm (PM_10_)) particulate matter (PM), black carbon (BC) and nitrogen dioxide (NO_2_) [1–4]. Out of these three, residential greenness has been associated with several positive health outcomes such as a lower adiposity level, higher birthweight and higher self-rated health [5–7], whereas the other two are linked with respiratory and cardiovascular disorders [1, 8–10]. Moreover, the combination of air pollution and smoking might increase the harmful effects [11].

Air pollution modelling is most often used to assess air pollution exposure [12, 13] and smoking behavior is typically self-reported in surveys [14, 15]. Although these models and surveys are validated, risks might be considerably underestimated because of exposure misclassification [16, 17]. For example, using the modelled air pollution at home is less reliable because: (1) depending on their mobility/behavior pattern, people are more often out of house than at home (work, hobbies, transport) [18], and (2) the type of ventilation and heating system might influence the indoor exposure [19, 20]. In the case of smoking behavior, research showed that self-reported smoking is often underestimated [21]. The use of biomarkers of exposure allows to improve personal exposure assessment as they represent the internal dose of an exposure [22]. Within this context, recent research developed a label-free and biocompatible method to measure BC in biological samples [23, 24]. Likewise, for accurate assessment of smoking status and behavior, cotinine and trans-3′-hydroxycotinine are used as metabolites of nicotine [25].

Although the effects of air pollution, green space and smoking have been studied extensively, still little is known about the pathways by which they affect human health. Especially in the case of mental health literature is limited. Some studies showed that access to green space is associated with an increase in physical activity and social cohesion, which in turn is positively associated with mental health [26–28]. On the other hand, one can also focus on the molecular pathways. To gain insight in the molecular process, biomarkers of effect can be used as they represent the early biological effect of an exposure before a disease occurs [22]. Mitochondrial DNA content (MtDNAc), a proxy for mitochondrial DNA copy number, and telomere length (TL) are both biomarkers that are very sensitive to and a target of reactive oxygen species (ROS) [29]. Air pollution and smoking might affect these biomarkers as the presence of particles in the lungs induce inflammation and ROS [30]. Additionally, mtDNAc and TL are considered biomarkers of biological aging. MtDNAc decreases with age [31] and telomere length shortens with every cell division [29]. These biomarkers have been associated with multiple age-related non communicable diseases (NCDs) like dementia, diabetes and hypertension [32]. Mental conditions such as depression, anxiety and bipolar disorder are more likely to co-occur in people with those age-related NCDs [33, 34], which suggests that mental health disorders might be associated with accelerated biological aging [35].

**Fig. 1:**
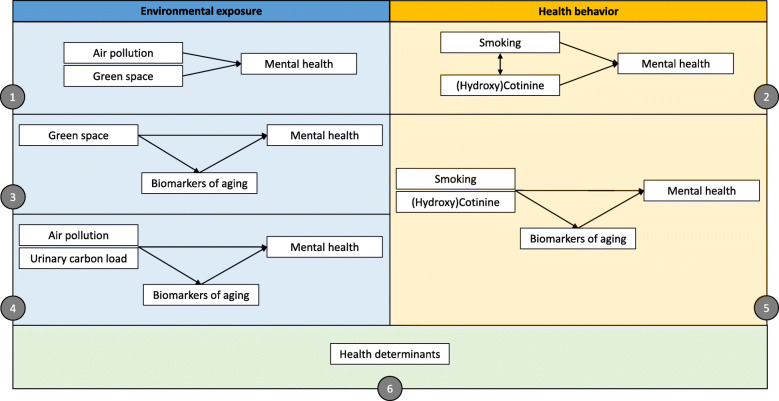
Graphic representation of the study design. Objectives are indicated in the figure by their appropriate number

The aim of this paper is to present the methods of the ongoing research project in which we evaluate the associations between environmental exposures (air pollution, residential green space), health behavior (smoking) and mental health using biomarkers of exposure and biological aging (Fig. 1).

More specifically, the objectives of the study, within a Belgian adult population, are:

1. To assess residential air pollution and green space in association with the mental health status (*n* = 11,611);
2. To assess smoking in association with the mental health status based on the smoking questionnaire data and the cotinine concentrations (n = 11,611 and 1168 respectively);
3. To perform a mediation analysis in which we evaluate if the biomarkers of biological aging are a mediator of the association between residential green space and the mental health status (*n* = 1168);
4. To perform a mediation analysis in which we evaluate if the biomarkers of biological aging are a mediator of the association between air pollution, represented by urinary BC, and the mental health status (*n* = 1168);
5. To perform a mediation analysis in which we evaluate if the biomarkers of biological aging are a mediator of the association between smoking, represented by cotinine, and the mental health status (n = 1168);
6. To assess which and how other health determinants (e.g. age, gender, socio-economic position, social health, physical activity, BMI, …) change the previously listed associations.

We hypothesize that air pollution and smoking have an adverse effect on the mental health status whereas residential green space induces the opposite effect. Furthermore, we hypothesize that part of these associations are mediated by the biomarkers of biological aging.

## Methods

### Study population

This study is part of the HuBiHIS (Human Biomonitoring as Added value of Health Interview Surveys) study. The objective of HuBiHIS is to evaluate the associations between environmental exposures, biomarkers of exposure and biological aging and three outcomes: respiratory, cardiovascular and mental health. In this part of the HuBiHIS study, the focus lies on mental health. The study population of HuBiHIS, and this cross-sectional study, is a subsample of the Belgian 2018 Health Interview Survey (BHIS). The BHIS is a comprehensive survey used to gain insight in the health status of the Belgian population. The survey takes place every four to five years and was last conducted between January 2018 and February 2019. The sampling frame is the National Register and participants are selected based on a multistage stratified sampling design [36]. Sampling weights are used for each individual taking into account age, gender, household size, province and quarter of the year in which the interview was done [37].

For the first time in 2018, a subsample was selected to participate in a supplementary Belgian Health Examination Survey (BELHES) [38]. Participants younger than 18 years, participants with a proxy respondent and people from the German community were not eligible for BELHES participation. Eligible BHIS respondents were invited from April 2018 onwards until a predefined number was obtained (*n* = 1100). All BELHES participants are included in the HuBiHIS subset except for those who did not provide either a blood or a urine sample. As part of the BELHES, this study was approved by the Medical Ethics Committee of the University Hospital Ghent (registration number B670201834895) and was carried out in line with recommendations of the Belgian Privacy Commission. All participants signed an informed consent before participation.

### Data sources and sample collection

#### Mental health indicators

The outcomes of this study are several mental health indicators including psychological and severe psychological distress, life satisfaction, vitality, eating disorders, suicidal ideation, subjective health and depressive and anxiety disorders, which can be derived from the BHIS. The BHIS interview is conducted by a trained interviewer during a home-visit. It consists of a face-to-face questionnaire, a self-administered questionnaire and a household questionnaire. The questions on mental health are part of the self-administered questionnaire and part of international standardized and validated questionnaires, which allow to calculate multiple mental health indicators (Table 1) [39]. (1) The general health questionnaire (GHQ-12) provides the prevalence of psychological distress in the population [40]. (2) Life satisfaction is scored based on the Cantril scale [41]. (3) The short form health survey (SF-36) gives an indication on the participants' vital energy level and is thus an indicator of the positive dimension of mental health [42]. (4) the SCOFF questionnaire is a 4-item questionnaire used to indicate eating disorders [43]. (5) The patient health questionnaire (PHQ-9) is a screening instrument for detecting major depression syndrome and other depression syndromes. (6) The general anxiety disorder questionnaire (GAD-7) provides the prevalence of general anxiety disorder across the population [44]. (7) Finally, a dichotomous question on suicidal ideation in the last 12 months and on subjective health is included.

**Table 1:** Description of the mental health indicators, used as outcomes, based on the questions in the Belgian Health Interview Survey 2018

Questionnaire	Indicator	Score	Use
**GHQ-12**	Indicator of mental well-being.	12 questions	1. 1. The sum (from 0 to 12) represents the global GHQ-score. The higher the score, the more change of physiological complaints.
		Answers: ‘better than usual’, ‘as good as usual’, ‘less than usual’, ‘much less than usual’	
			2. 1. A cut-off point of [2+] is used to identify those participants with at least 2 ‘abnormal’ psychological complaints, indicating a possible psychopathology.
		Ranking: [0 0 1 1]	
			3. A cut-off point of [4+] is used to identify those participants with at least 4 ‘abnormal’ psychological complaints, indicating the more severe cases.
**Cantril scale**	Life satisfaction	On a scale from 0 to 10, 0 indicating ‘completely dissatisfied’ and 10 indicating ‘completely satisfied’, how satisfied are you with your life?	1. The score indicates the life satisfaction. The higher the score, the more someone is satisfied with their life.
			2. The scale is divided into three groups: 0 = low satisfaction (0–5), 1 = average satisfaction (6–8), 2 = high satisfaction (9–10).
**SF-36**	Indicator of the positive psychological health (vital energy)	4 questions	1. 1. The average of the scores is converted to a scale from 0 to 100. The higher the score, the higher the vitality.
		Answers: ‘always’, ‘most of the time’, ‘sometimes’, ‘rarely’, ‘never’	
		Ranking: [5 4 3 2 1] for the first two questions and [1 2 3 4 5] for the last two questions.	2. A dichotomous score indicates those who have an energy and vitality well above average (calculated as the average of all participants + the standard deviation)
**SCOFF**	Indicator in regard to possible eating disorders	5 yes or no questions	Participants are identified as having an eating disorder when they answered at least twice ‘yes’.
**PHQ-9**	Indicator of a depressive disorder and depression severity score	9 questions	1. 1. A dichotomous indicator is used to identify who suffers from major depression syndrome (MDD).
		Answers: ‘not at all’, ‘several days’, ‘more than half the days’ and ‘nearly every day’	
			2. A dichotomous indicator is used to identify who suffers from any type of depression other than MDD.
			3. The combination of both is used to identify participants with any type of depression.
			4. To indicate the severity score, the participants are divided into five groups: 0 = no depression, 1 = mild depression, 2 = moderate depression, 3 = moderate/severe depression, 5 = severe depression.
**GAD-7**	Indicator of general anxiety disorder and anxiety severity score	7 questions	1. A cut-off point of [10+] is used to identify who suffers from a general anxiety disorder.
		Answers: ‘not at all’, ‘several days’, ‘more than half the days’ and ‘nearly every day’	2. 1. To indicate the severity score, the participants are divided into four groups: 0 = no anxiety, 1 = mild anxiety, 2 = moderate anxiety, 3 = severe anxiety.
		Ranking: [0 1 2 3]	

GHQ-12, General health questionnaire; SF-36, Short form health survey; PHQ-9, Patient health questionnaire; GAD-7, Anxiety disorder questionnaire

#### Smoking status

The BHIS is also used to gain knowledge on the smoking behavior of the Belgian population. The indicator used in this study makes a distinction between daily smokers, occasional smokers, ex-smokers and persons who never smoked. Finally, general characteristics including age, gender, ethnicity, BMI, passive smoking, socio-economic status, social health and alcohol use can be derived from the BHIS.

#### Residential air pollution and green space exposure

Residential addresses of the participants are geocoded. The daily concentrations to nitrogen dioxide (NO_2_), particulate matter ≤2.5 μm (PM_2.5_) and ≤ 10 μm (PM_10_) and black carbon (BC) are modelled with a high resolution using a spatial temporal interpolation model. This model uses land cover data obtained by satellite imagery (CORINE land-cover data set) [45] and pollution data provided by the Belgian fixed monitoring stations in combination with a dispersion model, including point and line sources [46, 47]. We calculate the average residential exposure during the year before BHIS participation as well as the exposure during the day, week, month and year before BELHES participation.

Residential green space is defined with a Geographic Information System (GIS). Two methods are used to calculate green space exposure. Firstly, the Groenkaart Vlaanderen 2012 (Green Map of Flanders), generated by the Agency for Geographic Information Flanders (AGIV), divides Flanders land cover into “no green”, “agriculture”, “low green” (vegetation < 3 m, including non-agricultural grasslands and meadows) and “high green” (vegetation > 3 m) areas [48]. Secondly, using the CORINE land cover 2012 (European Environment Agency), the extent of semi-natural and forested-, agricultural-, residential and industrial area is calculated. Green space is calculated in a radius of 50 m, 100 m, 300 m, 500 m, 1000 m, 2000 m and 5000 m around the participants’ residence. ArcGIS 10 software is used for all GIS analyses.

#### Biomarkers of exposure and biological aging

After participation to the BHIS, participants are, if they are eligible, asked if they are willing to participate to the BELHES. If so, a trained nurse visits to conduct a small questionnaire and to take multiple anthropometric measurements and a spot urine and blood (EDTA) sample. These samples are used to measure respectively the biomarkers of exposure and biological aging. Before storage, the EDTA tube is centrifuged to facilitate future DNA extraction using the buffy coat.

Black carbon particles are measured in urine using a biocompatible label-free detection method by a femtosecond pulsed laser microscopy according to a validated protocol [23, 49]. Cotinine and trans-3′-hydroxycotinine are measured by on-line solid-phase extraction with ultra-performance liquid chromatography and tandem mass spectrometry (SPE-UPLC MS/MS) as described previously [50].

For the biomarkers of biological aging, DNA is extracted from buffy coat using the QIAgen Mini Kit (Qiagen, N.V.V Venlo, The Netherlands). The purity of the sample is measured with a NanoDrop spectrophotometer (ND-2000; Thermo Fisher Scientific, Wilmington, DE, U.S.A.). DNA quantity is assessed with a Quant-iT™ PicoGreen® dsDNA Assay Kit (Life Technologies, Foster City, CA, USA). DNA integrity is assessed by agarose gel-electrophoresis. Mean relative TL and mtDNAc (ratio of telomere gene (telg/telc) copy number or mitochondrial gene (MT-ND1) copy number to a reference gene (HBG1)) are measured in triplicate using a previously described modified quantitative real-time PCR (qPCR) assay [51, 52]. All measurements are performed on a 7900HT Fast Real-Time PCR System (Applied Biosystems) in a 384-well format. Inter-run calibrators (IRCs) are used to account for inter-run variability. Also negative controls are used in each run. Raw data is processed and normalized to the reference gene using qBase plus software (Biogazelle, Zwijnaarde, Belgium).

### Statistical analysis

Statistical analysis is done with SAS 9.3 (SAS Institute, Cary, NC). Analyses are adjusted for a priori selected confounders and covariates including age, gender, ethnicity, BMI, passive smoking, socio-economic status and season, depending on their contribution to the model. Additionally, to account for the complex study design, weighting, clustering and stratification are included in the model. To address the first and second objective, multivariable linear or logistic regressions are used depending on the mental health indicator. A mediation analysis developed by Valeri et al. (2013) is used to assess the third, fourth and fifth objective. The ratio of the indirect effect and the direct effect will show the proportion of mediation by the biomarkers of biological aging [53]. Finally, population attributable fractions (PAF’s) are used to identify which personal characteristics are associated with the different mental health indicators [54, 55].

## Results

**Fig. 2:**
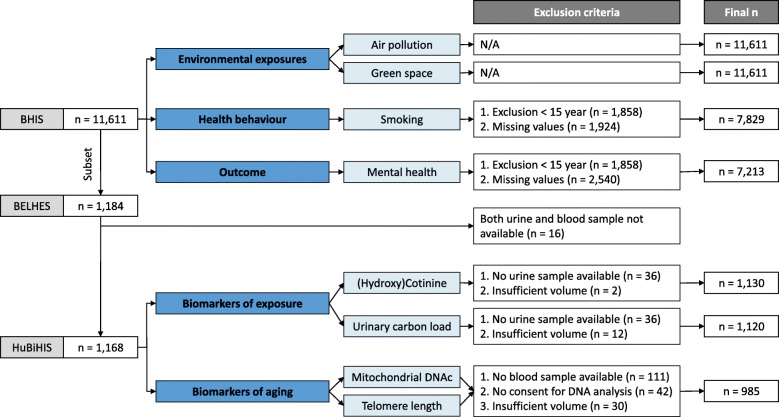
The data collection of the outcome, exposures and biomarkers with the exclusion criteria and the final number of participants for each section. Mitochondrial DNAc = Mitochondrial DNA content

In 2018, Belgium had a total of 11,376,070 residents. 5692 households participated to the BHIS, resulting in a household participation rate of 57.5% and a total of 11,611 BHIS participants. Of all eligible BHIS participants that were contacted, 1184 participated to the BELHES, indicating a participation rate of 24.1% [38]. 1.4% of those participants provided neither a blood nor a urine sample and were excluded from the HuBiHIS study, which ended up in a final subset of 1168 participants. Figure 2 shows an overview of the different outcomes, biomarkers and exposures and the number of respondents for which the data is available. Firstly, air pollution exposure and residential green space is available for all BHIS participants. Secondly, the questions on the smoking and mental health status in the BHIS are answered by respectively 7829 and 7213 BHIS participants. For 7096 participants both the smoking and the mental health status are available. Finally, biomarker assessment is performed on blood and urine samples of the HuBiHIS participants. 96.9% of the HuBiHIS participants provided a urine sample. Because 1.1% did not provide a sufficient volume, urinary (hydroxy) cotinine and BC levels are measured for respectively 1130 and 1120 participants. 99.0% of the HuBiHIS participants provided a blood sample. However, 4.0% did not provide informed consent for DNA analysis. Also, not all samples are suitable for DNA extraction. Insufficient volumes reduce the amount of DNA samples to 985. Finally, there are 820 participants for which all data in the HuBiHIS dataset is available.

As participation was on a voluntary basis a selection bias is quite likely. An overview of the main characteristics of the Belgian population and the BHIS and HuBiHIS participants is presented in Table 2. Only a small deviation in gender is observed. The difference in age between the BHIS and the HuBiHIS participants is because only adults are included in the latter, which increases the average age. The biggest difference in participation is shown for adults between 25 and 64 years whereas the category of 65+ remains constant. Education and income are two determinants of socio-economic status. Of all HuBiHIS participants, most have a college or university degree. Also, HuBiHIS participants, who all participated to the BELHES, have a significantly higher education (*p* < 0.0001) and income (*p* < 0.0001) level compared to BHIS participants who did not participate to the BELHES. This indicates that there is a possible selection bias as people with a higher socio-economic status were more willing to participate to the BELHES.

**Table 2:** Characteristics of the Belgian population in 2018 and the BHIS and HuBiHIS subset. Mean (SD) or n (%)

	Belgium	BHIS	HuBiHIS
**Age**	NA	43.0 (23.6)	50.1 (16.3)
**15–24 years**	1,298,448 (11.4%)	1059 (9.1%)	66 (5.7%)
**25–44 years**	2,945,994 (25.9%)	2916 (25.1%)	393 (34.0%)
**54–64 years**	3,073,332 (27.0%)	3395 (29.2%)	458 (39.2%)
**65+ years**	2,130,556 (18.7%)	2383 (20.6%)	251 (21.5%)
**Gender**			
**Female**	5,778,164 (50.8%)	6023 (51.9%)	614 (52.6%)
**Male**	5,597,906 (49.8%)	5588 (48.1%)	554 (47.4%)
**Education**^**a**^			
**Up to primary school**	NA	2369 (27.6%)	233 (21.6%)
**Up to secondary school**	NA	2750 (32.1%)	353 (32.8%)
**College or university degree**	NA	3461 (40.3%)	492 (45.6%)
**Household income**^**b**^	NA	2665.4 (1634.1)	2942.0 (1739.0)

^a^ data available for 8580 and 1078 participants respectively

^b^ data available for 4676 and 660 participants respectively

## Discussion

Multiple studies evaluated the effects of the environment and health behavior on respiratory and cardiovascular morbidity [2, 9, 56]. Recently, studies also found these associations for mental health. Exposure to residential greenness and green space was positively associated with mental health outcomes [57, 58] whereas the opposite was seen for smoking and air pollution [59–61]. Also TL and mtDNAc have been associated with these exposures [62–64] and with several mental health disorders [65, 66]. However, to our knowledge, this is the first research that combines these exposures, biomarkers and health outcomes.

The use of biomarkers is the main strength of this study. Biomarkers are defined by the US National Research Council (NRC) as “… a change induced by a contaminant in the biochemical or cellular components of a process, structure or function that can be measured in a biological system” [67]. In this case, using biomarkers of exposure to measure exposure to air pollution and smoking will result in a lower variance, making the results more reliable compared to the use of air samplers or surveys [68]. Furthermore, measuring BC in urine is done with a new and patented technique, which has been shown to represent medium-term to chronic exposure to traffic-related air pollution [23, 49]. TL and mtDNA on the other hand are known as the main biological mediators in the core axis of aging [69]. They are affected by multiple personal characteristics and external exposures. As such, they might explain the intermediate pathway in which air pollution, residential green space and smoking affect human health [29]. A second strength is the amount of data that is obtained. The BHIS 2018 questionnaire is an extensive survey with a large amount of information on health and health behavior [39]. Finally, this study is one of the first to provide insight on the association between the environment and mental health disorders on a large study population in Belgium. Also, the results of the study can provide insights on the health of the Belgian population, making it a nationwide interesting study.

We also acknowledge some limitations of the study. Cross-sectional studies have the common limitation that they take a single moment in time and therefore miss the change within the individual over time. Especially with the use of biomarkers of biological aging it would be interesting to see the change after a given period of time. Furthermore, because of this study design it is not possible to identify the direction of the effect but only to identify associations. A second limitation is that there is a distinct time lag between the execution of the BHIS questionnaire and the BELHES sample collection. This time lag ranges from a 5 to 221 days and is different for most participants. Consequently, associations between data derived from both the BELHES and BHIS become more challenging. Finally, as multiple BHIS questionnaires are self-administered, we expect quite a lot of missing values. Due to privacy regulations, we cannot re-contact the participants. Also, it was not possible to acquire more information (such as address) on the participants’ workplace. Ambient air pollution concentrations are modelled at the participants’ residence, even though people spend a considerable amount of time at or on their way to work. This might result in exposure misclassification. Nevertheless, this research project explores the use of biomarkers of exposure in order to mitigate this limitation.

## Conclusion

In this cross-sectional study we aim to assess the association between environmental exposures (air pollution, residential green space), health behavior (smoking) and mental health. To obtain the most reliable results and to gain knowledge on the molecular pathways of the effects, biomarkers of respectively exposure and biological aging are used.

## Data Availability

The dataset used for this study is available through a request to the Health Committee of the Data Protection Authority.

## Abbreviations

BC: Black Carbon
GAD-7: General Anxiety Disorder questionnaire
GIS: Geographic Information System
GHQ-12: General Health Questionnaire
BELHES: Belgian Health Examination Survey
BHIS: Belgian Health Interview Survey
HuBiHIS: Human Biomonitoring as Added value of Health Interview Surveys
IRC: Interrun calibrator
MDD: Major Depression Syndrome
mtDNA: Mitochondrial DNA
mtDNAc: Mitochondrial DNA Content
NCD: Non communicable disease
NO_2_: Nitrogen Dioxide
NRC: US National Research Council
PAF: Population Attributable Fraction
PHQ-9: Patient Health Questionnaire
PM_2.5_: Particulate matter with a diameter < 2.5 μm
PM_10_: Particulate matter with a diameter < 10 μm
ROS: Reactive Oxygen Species
qPCR: Quantitative real-time Polymerase Chain Reaction
SF-36: Short Form health survey
TL: Telomere Length
